# Mortality rate of percutaneous coronary interventions in ST-segment elevation myocardial infarction patients under the public health insurance schemes of Thailand

**DOI:** 10.3389/fcvm.2024.1397015

**Published:** 2024-10-04

**Authors:** Suppavit Chumsantivut, Somrat Lertmaharit, Thanapoom Rattananupong, Vorarit Lertsuwunseri, Siriporn Athisakul, Chaisiri Wanlapakorn, Suphot Srimahachota

**Affiliations:** ^1^ Department of Preventive and Social Medicine, Faculty of Medicine, Chulalongkorn University, Bangkok, Thailand; ^2^ Cardiac Center and Division of Cardiovascular Disease, Department of Medicine, King Chulalongkorn Memorial Hospital, Chulalongkorn University, Bangkok, Thailand

**Keywords:** STEMI, ST-elevation myocardial infarction, healthcare, percutaneous coronary intervention, Thailand, mortality, PCI

## Abstract

**Background:**

In Thailand, access to specific pharmaceuticals and medical devices for ST-elevation myocardial infarction (STEMI) patients is restricted within certain healthcare systems, leading to inequalities in the quality of medical care among different healthcare systems. This study aims to compare mortality rates within one year of STEMI patients among the public health insurance schemes of Thailand.

**Methodology:**

This study is a single-center retrospective analysis of patients with STEMI treated with primary percutaneous coronary intervention (pPCI). It involves patients utilizing various state health insurance schemes in Thailand from January 1, 2010, to December 31, 2020. Data collection occurred through the hospital's computerized management system and the registration administration office of the Department of Provincial Administration.

**Results:**

The study involved 1,077 patients, categorized into three groups based on their state health insurance: Universal Health Coverage (UC) (546 patients, 50.7%), Social Security System (SS) (199 patients, 18.5%), and Civil Service Reimbursement (CS) (332 patients, 30.8%). The one-year mortality rates in these groups were 10.57%, 4.21%, and 6.47%, respectively (*p* = 0.010). In the unadjusted model, the SS group showed a lower risk of one-year mortality [Hazard Ratio (HR) 0.38, 95% CI 0.18–0.80, *p* = 0.011], and the CS group also demonstrated a lower risk (HR 0.59, 95% CI 0.35–0.99, *p* = 0.047) compared to the UC group. In the adjusted model, only the CS group significantly reduced the risk of one-year mortality. Other factors that affected one-year mortality were age ≥65 years, prior coronary artery diseases, Killip class 3–4, pre-discharge prescription of angiotensin-converting enzyme inhibitors, occlusion in the left anterior descending artery, multivessel disease, in-hospital atrial fibrillation/flutter and in-hospital pericardial effusion.

**Conclusion:**

Healthcare schemes play a significant role in influencing one-year mortality rates among STEMI patients treated with pPCI. This information would be crucial for developing strategies and programs to aid healthcare policymakers at both regional and international levels in reducing morbidity and mortality.

## 1 Introduction

ST-segment elevation myocardial infarction (STEMI) presents a major public health challenge worldwide, including in Thailand (1). The prevalence of STEMI within the nation exhibits an ascending trend, leading to significant rates of morbidity and mortality (2–4). As recommended by international guidelines, primary percutaneous coronary intervention (pPCI) is currently the preferred therapeutic intervention for STEMI patients when performed by experts within an optimal timeframe (5, 6). Compared with traditional intervention, thrombolytic therapy, pPCI demonstrates superior efficacy, significantly reducing short-term and long-term mortality rates, the incidence of recurrent arterial occlusions, the risk of cerebrovascular events, and the occurrence of abnormal bleeding (7).

In Thailand, most healthcare services are predominantly provided by the government sector. Healthcare services in Thailand are divided into three distinct systems: Universal Health Coverage (UC), Social Security System (SS), and Civil Service Reimbursement (CS), which cover 75%, 16%, and 9% of the population, respectively. The UC's objective is to provide healthcare services without incurring personal financial costs for Thai citizens. The SS is intended for private sector employees, providing a safety net for life sustenance through contributions from employers, employees, and the state. The CS is a privilege for active and retired civil servants, representing one of the welfare benefits provided by the government (8).

Each system has its own conceptual underpinnings, leading to inequalities in public health service access among the three types of health coverage. This results in discrepancies in access to medications and variations in the quality of treatments, leading to a potential significant impact on the quality of life of patients, both in the short and long term. The authors sought to determine the differences in patients' characteristics, procedure variations, in-hospital outcomes, and long-term outcomes among the healthcare systems. Therefore, this study aims to compare mortality rates within one year of STEMI patients among the public health insurance schemes of Thailand. This information would be crucial for developing strategies and programs to aid healthcare policymakers at both regional and international levels in reducing morbidity and mortality.

## 2 Methods

### 2.1 Study design

This study is a descriptive, retrospective research on patients diagnosed with ST-elevation myocardial infarction (STEMI), who received primary percutaneous coronary intervention (pPCI), at the King Chulalongkorn Memorial Hospital, a tertiary referral center. The data for this study was compiled from the King Chulalongkorn Memorial Hospital's database management system, utilizing International Classification of Diseases, Tenth Revision, Clinical Modification codes I21.0–I21.4, and the Ninth Revision, Clinical Modification codes 00.40–0048, 00.6, and 36.06–36.07. The period under review in this study extends from January 1, 2010, through December 31, 2020.

We identified and recorded the total number of patients who met the pre-defined inclusion criteria: patients who received the pPCI strategy as the initial treatment, and whose age was equal to or greater than 18 years. We excluded patients with missing data on national identification number, health insurance coverage under the Thai national health insurance scheme, or no history in the civil registration system, patients who received the pharmacoinvasive strategy, and patients who underwent coronary artery bypass grafting (CABG) surgery. Patients were followed for mortality from the time of procedure initiation. However, the analysis exclusively included deaths that occurred post-procedure. Demographic and clinical information, including laboratory test results and procedural data, were retrospectively collected by well-trained critical care nurses and cardiologists, and documented from both outpatient and inpatient database records. Routine follow-up data collection included hospital visits and assessments at 12 months via file review for specific clinical measures, patient-reported outcomes, or hospitalizations.

For verifying the timing of deaths, our study was granted access to the civil registration system through the Bureau of Registration Administration (BORA) of Thailand. We performed a matching process using the patient's national identification number and date of birth against entries in the civil registration system. A match was confirmed when an entry in the Index corresponded exactly to a patient's national identification number and date of birth with a patient recorded in our study registry. ChatGPT 4.0 is applied to check and correct grammatical errors during writing process. The contents produced by AI technology were finally reviewed and edited by the authors.

This study was conducted in adherence to the principles of Good Clinical Practice, adhering to all relevant Thai legislation and data protection regulations. No individual specific consent forms for the study were obtained. The protocol was reviewed and approved by an ethics committee from the Institutional Review Board of the Faculty of Medicine, Chulalongkorn University, Bangkok, Thailand (COA No. 1742/2022).

### 2.2 Statistical analysis

The primary objective is to compare mortality rates within one year of STEMI patients after treatment of pPCI among the public health insurance schemes of Thailand. The secondary objectives are to study prognostic determinants for both in-hospital and one-year mortality rates among patients diagnosed with STEMI who have undergone pPCI at King Chulalongkorn Memorial Hospital.

Categorical variables are presented as frequency and proportion using the Pearson Chi-square test or Fisher's exact test, while continuous variables are expressed through mean, standard deviations (SD), medians, or interquartile ranges (IQR) analyzed with the independent *t*-test, one-way analysis of variance (ANOVA), or Kruskal-Wallis test. The study aims to conduct a comparative analysis of one-year mortality rates across different healthcare entitlements in Thailand, including the Universal Health Coverage, Social Security System, and Civil Service Reimbursement. To assess differences in mortality outcomes among these groups, Kaplan-Meier curve and log-rank testing methodologies are utilized. Multiple logistic regression analyses were executed to determine the predictors of in-hospital outcomes, while multiple Cox regression models to compute crude hazard ratios are utilized to examine the individual relation between each predictor and death during one-year follow-up. Variables selected for inclusion in the final multivariate models were those demonstrating a significance level of less than 0.05 in the univariate analyses (Supplementary Tables S1, S2), unless otherwise stated. All statistical tests were conducted as two-sided, with the threshold for statistical significance established at a *P*-value of less than 0.05. All statistical analyses will be performed using StataCorp. 2023. Stata Statistical Software: Release 18. College Station, TX: StataCorp LLC.

## 3 Results

**Figure 1:**
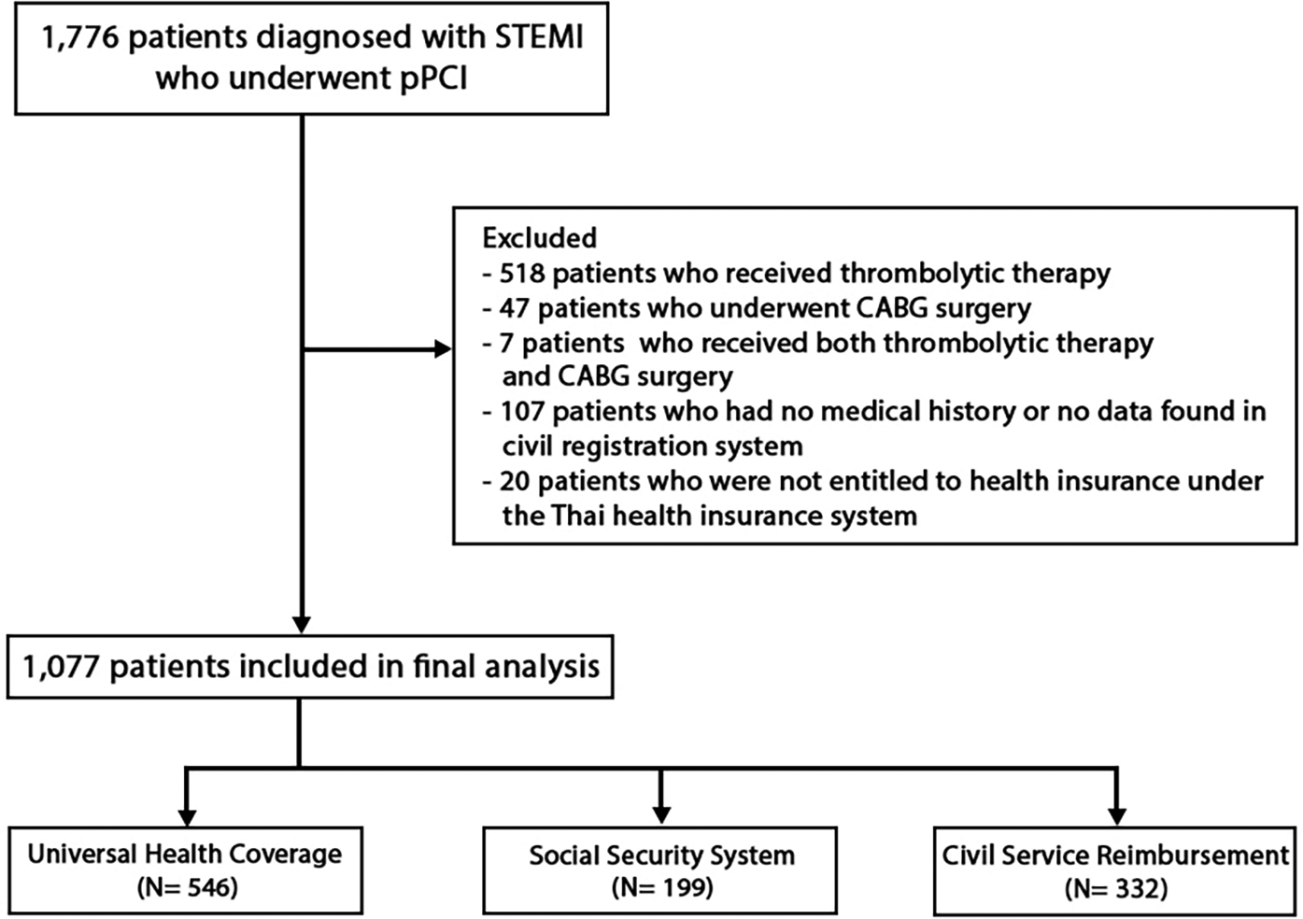
Flowchart of cohort selection.

In this study, we examined data from 1,776 STEMI patients who underwent PCI for STEMI from January 1, 2010, to December 31, 2020. Following the application of exclusion criteria, which led to the removal of 699 patients as depicted in Figure 1, our analysis focused on the remaining 1,077 participants. These individuals were stratified into three distinct groups based on their enrollment in public health insurance schemes of Thailand: 546 patients in the Universal Health Coverage (UC) group (50.69%), 199 patients in the Social Security System (SS) group (18.48%), and 332 patients in the Civil Service Reimbursement (CS) group (30.83%). Baseline demographic and clinical characteristics, presented in Table 1, reveal that the SS cohort exhibited a higher male predominance (86.90%), the youngest mean age (51.62 years), and the most significant proportion of transferred patients (71.90%). Notably, this group also reported the highest proportion of Killip class I at hospital admission (79.90%). Laboratory findings showed the lowest mean serum creatinine levels in the SS group at 0.945 mg/dl and the lowest LDL levels in the CS group at 121 mg/dl.

**Table 1:** Baseline characteristics of STEMI treated with pPCI.

Variables	UC (*N* = 546)	SS (*N* = 199)	CS (*N* = 332)	Total patients (*N* = 1,077)	*p*-value
Male gender, *n* (%)	396 (72.53)	173 (86.93)	234 (70.48)	803 (74.56)	<0.001^†^
Mean age (SD)	61.04 (12.7)	51.62 (9.1)	64.91 (14.7)	60.49 (13.6)	<0.001^‡^
Group by age, *n* (%)					<0.001^†^
<50 years	97 (17.77)	84 (42.21)	48 (14.46)	229 (21.26)	
50–59 years	163 (29.85)	77 (38.69)	94 (28.31)	334 (31.01)	
60–69 years	156 (28.57)	32 (16.08)	67 (20.18)	255 (23.68)	
70–79 years	88 (16.12)	6 (3.02)	58 (17.47)	152 (14.11)	
>79 years	42 (7.69)	0 (0.00)	65 (19.58)	107 (9.94)	
Refer case, *n* (%)	350 (64.10)	143 (71.86)	181 (54.52)	674 (62.58)	<0.001^†^
Median refer time (IQR) (Q1, Q3)	45 (30) (35, 65)	35 (20) (30, 50)	65.5 (41) (50, 91)	46 (35) (35, 70)	<0.001^§^
Mean body mass index (SD)	24.06 (3.93)	25.06 (3.79)	24.45 (3.66)	24.37 (3.84)	0.009^‡^
Group by weight, *n* (%)					0.055^†^
Underweight (<18.5 kg)	32 (5.86)	5 (2.51)	10 (3.01)	47 (4.36)	
Normal (18.5–22.9 kg)	173 (31.68)	50 (25.13)	88 (26.51)	311 (28.88)	
Overweight (23.0–24.9 kg)	119 (21.79)	54 (27.14)	84 (25.30)	257 (23.86)	
Obese (>24.9 kg)	222 (40.66)	90 (45.23)	150 (45.18)	462 (42.90)	
Cardiovascular risk factors, *n* (%)					
Diabetic mellitus	213 (39.01)	67 (33.67)	118 (35.54)	398 (36.95)	0.333^†^
Hypertension	349 (63.92)	90 (45.23)	227 (68.37)	666 (61.84)	<0.001^†^
Dyslipidemia	418 (76.56)	158 (79.40)	275 (82.83)	851 (79.02)	0.085^†^
Coronary artery diseases	58 (10.62)	13 (6.53)	44 (13.25)	115 (10.68)	0.052^†^
Cerebrovascular diseases	47 (8.61)	4 (2.01)	31 (9.34)	82 (7.61)	0.004^†^
Atrial fibrillation/flutter	17 (3.11)	2 (1.01)	7 (2.11)	26 (2.41)	0.230^†^
Chronic kidney disease	52 (9.52)	8 (4.02)	41 (12.35)	101 (9.38)	0.006^†^
Heart failure	17 (3.11)	1 (0.50)	5 (1.51)	23 (2.14)	0.059^†^
Alcohol status, *n* (%)					0.076^†^
Current	89 (18.50)	39 (21.67)	56 (18.48)	184 (19.09)	
Previous	58 (12.06)	25 (13.89)	39 (12.87)	122 (12.66)	
Social	46 (9.56)	26 (14.44)	20 (6.60)	92 (9.54)	
Smoking status, *n* (%)					<0.001^†^
Current	211 (42.20)	99 (51.83)	91 (29.35)	401 (40.06)	
Previous	74 (14.80)	25 (13.09)	61 (19.68)	160 (15.98)	
Median blood pressure and heart rate at admission (IQR) (Q1, Q3)					
Systolic blood pressure	126 (48) (100, 148)	126 (42) (102, 144)	127 (40) (110, 150)	126 (45) (103, 148)	0.644^§^
Diastolic blood pressure	78 (30) (60, 90)	77.5 (26) (65, 91)	78 (25) (62, 87)	78 (29) (61, 90)	0.556^§^
Heart rate	77 (28) (62, 90)	78 (30) (60, 90)	80 (27) (63, 90)	78 (28) (62, 90)	0.638^§^
Arrest at admission, *n* (%)	45 (8.24)	16 (8.04)	19 (5.72)	80 (7.43)	0.361^†^
Diagnosis-to-wire time <90 min, *n* (%)	493 (90.62)	188 (94.95)	287 (86.71)	968 (90.21)	0.008^†^
Diagnosis-to-Wire time <120 min (Referred), *n* (%)	184 (48.17)	81 (55.10)	62 (30.24)	327 (44.55)	<0.001^†^
Killip class, *n* (%)					0.019^†^
I	361 (66.12)	159 (79.90)	229 (68.98)	749 (69.55)	
II	61 (11.17)	11 (5.53)	30 (9.04)	102 (9.47)	
III	31 (5.68)	4 (2.01)	16 (4.82)	51 (4.74)	
IV	93 (17.03)	25 (12.56)	57 (17.17)	175 (16.25)	
Lipid profile, mg/dl					
Mean total cholesterol (SD)	192.86 (52.97)	204.21 (52.77)	191.39 (59.11)	194.50 (55.08)	0.027^‡^
Mean HDL (SD)	40.53 (12.08)	39.96 (10.80)	41.76 (12.00)	40.81 (11.84)	0.194^‡^
Median LDL (IQR) (Q1, Q3)	126 (97, 159) (62)	140 (104, 167) (63)	121 (94, 154) (60)	128 (96, 159) (63)	0.002^§^
Median triglyceride (IQR) (Q1, Q3)	108 (77, 148) (71)	115 (82, 189) (107)	109 (76, 148) (72)	109 (78, 158) (80)	0.012^§^
Median peak serum creatinine, mg/dl (IQR) (Q1, Q3)	1.07 (0.69) (0.85, 1.54)	0.945 (0.34) (0.81, 1.15)	1.04 (0.66) (0.85, 1.51)	1.04 (0.57) (0.84, 1.41)	<0.001^§^
Median HbA1C (IQR) (Q1, Q3)	5.70 (5.3, 6.8) (1.5)	5.70 (5.3, 6.6) (1.3)	5.70 (5.4, 6.6) (1.2)	5.70 (5.3, 6.7) (1.4)	0.707^§^

CS, Civil Service Reimbursement; SS, Social Security System; UC, Universal Health Coverage.

^†^ Chi-square test.

‡ One-way analysis of variance.

^§^ Kruskal-Wallis test.

Significant statistical differences between groups were observed in hospital complications (Table 2), including cardiac arrest with non-shockable rhythm, atrial fibrillation/atrial flutter, heart failure, acute kidney injury, and infections. Remarkably, these complications were least prevalent in the SS group, with in-hospital mortality also lowest in this cohort. Through multiple logistic regression analysis (Table 3), variables for in-hospital mortality were identified, including age more than or equal to 65, cardiac arrest upon admission, advanced Killip class 3–4, multivessel disease, left main artery occlusion, alongside complications such as bradycardia, pericardiac effusion, atrial fibrillation/flutter, ventricular tachycardia, and acute kidney failure. In contrast, the use of drug-eluting stents represented a protective factor against in-hospital mortality.

**Table 2:** In-hospital complications of STEMI treated with pPCI.

Variables	UC (*N* = 546)	SS (*N* = 199)	CS (*N* = 332)	Total patients (*N* = 1,077)	*p*-value
Cardiac arrest with non-shockable rhythm	51 (9.34)	8 (4.02)	22 (6.63)	81 (7.52)	0.039^†^
Atrial fibrillation/Atrial flutter	66 (12.09)	13 (6.53)	44 (13.25)	123 (11.42)	0.049^†^
Bradycardia	144 (26.37)	59 (29.65)	83 (25.00)	286 (26.56)	0.497^†^
2-degree atrioventricular block	7 (1.28)	2 (1.01)	9 (2.71)	18 (1.67)	0.199^†^
Complete atrioventricular block	40 (7.33)	14 (7.04)	31 (9.34)	85 (7.89)	0.498^†^
Ventricular tachycardia	33 (6.04)	4 (2.01)	14 (4.22)	51 (4.74)	0.062^†^
Heart failure	256 (46.89)	56 (28.14)	147 (44.28)	459 (42.62)	<0.001^†^
Cardiogenic shock	139 (25.46)	34 (17.09)	77 (23.19)	250 (23.21)	0.057^†^
Acute kidney injury	98 (17.95)	17 (8.54)	54 (16.27)	169 (15.69)	0.007^†^
Infection	81 (14.84)	16 (8.04)	40 (12.05)	137 (12.72)	0.044^†^
Hematoma	74 (13.55)	21 (10.55)	38 (11.45)	133 (12.35)	0.455^†^
Gastrointestinal bleeding	53 (9.71)	11 (5.53)	27 (8.13)	91 (8.45)	0.187^†^
Mean hospitalization duration, days (SD)	3.75 (8.14)	2.36 (4.09)	4.12 (6.64)	3.61 (7.11)	0.018^§^
In-hospital death	54 (9.89)	9 (4.52)	23 (6.93)	86 (7.99)	0.040^†^
Cardiac death	50 (92.59)	9 (100)	21 (91.30)	80 (93.02)	1.000^‡^
Non-cardiac death	4 (7.41)	0 (0.00)	2 (8.70)	6 (6.98)	
Death in catheterization room	7 (1.28)	1 (0.50)	1 (0.30)	9 (0.84)	0.330^‡^

CS, Civil Service Reimbursement; SS, Social Security System; UC, Universal Health Coverage.

^†^ Chi-squared test.

^‡^ Fisher-exact test.

^§^ One-way analysis of variance.

**Table 3:** Multiple logistic analysis of predictors of in-hospital mortality after pPCI.

Variables	Odds ratio	95% Confidence interval	*p*-value
Age ≥ 65 years old	1.97	1.05–3.70	0.035
Cardiac arrest at admission	5.57	2.69–11.53	<0.001
Killip class 3–4	6.07	3.11–11.86	<0.001
Pericardial effusion	16.09	3.67–70.53	<0.001
Bradycardia	2.72	1.47–5.02	0.001
Atrial fibrillation/flutter	2.25	1.15–4.42	0.018
Ventricular tachycardia	5.31	2.31–12.18	<0.001
Acute kidney failure	2.87	1.50–5.48	0.001
Multivessel disease	3.36	1.55–7.28	0.002
Left main artery	5.24	1.64–16.77	0.005
Drug-eluting stent	0.49	0.26–0.93	0.029

In terms of cardiac catheterization procedures, as presented in Table 4, the SS group had the highest proportion of procedures completed within the Diagnosis-to-Wire time of less than 90 min (94.95%), and also achieved the highest proportion within <120 min (55.10%) drug-eluting stents (DES) appeared to be the predominant choice (78.92%), followed by plain old balloon angioplasty (POBA) and bare-metal stents (BMS) at 17.08% and 8.91%, respectively. The SS cohort led in DES utilization (85.43%), with the UC and CS groups behind at 80.95% and 71.69%, respectively.

**Table 4:** Procedure variables of STEMI treated with pPCI.

Variables, *n* (%)	UC (*N* = 546)	SS (*N* = 199)	CS (*N* = 332)	Total patients (*N* = 1,077)	*p*-value
Pacemaker	43 (7.88)	11 (5.53)	25 (7.53)	79 (7.34)	0.546^†^
Mechanical circulatory supports	90 (16.48)	26 (13.07)	51 (15.36)	167 (15.51)	0.520^†^
Swan Ganz catheter	28 (5.13)	5 (2.51)	13 (3.92)	46 (4.27)	0.274^†^
Endotracheal intubation	123 (22.53)	23 (11.56)	56 (16.87)	202 (18.76)	0.002^†^
Left ventricular ejection fraction					0.002^†^
**≤**40%	174 (32.95)	37 (18.97)	81 (24.62)	292 (27.76)	
40–50%	97 (18.37)	47 (24.10)	65 (19.76)	209 (19.87)	
≥50%	257 (48.67)	111 (56.92)	183 (55.62)	551 (52.38)	
Entry location					0.026^‡^
Femoral	513 (93.96)	176 (88.44)	315 (94.88)	100 (93.22)	
Radial	30 (5.49)	22 (11.06)	17 (5.12)	69 (6.41)	
Brachial	3 (0.55)	1 (0.50)	0 (0.00)	4 (0.37)	
Number of vessel disease					0.013^†^
Single vessel disease	196 (35.90)	79 (39.70)	136 (40.96)	411 (38.16)	
Double vessel disease	148 (27.11)	71 (35.68)	94 (28.31)	313 (29.06)	
Triple vessel disease	202 (37.00)	49 (24.62)	102 (30.72)	353 (32.78)	
Type of stent					
Bare-metal stent	33 (6.04)	9 (4.52)	54 (16.27)	96 (8.91)	<0.001^†^
Drug-eluting stent	442 (80.95)	170 (85.43)	238 (71.69)	850 (78.92)	<0.001^†^
Plain old balloon angioplasty	105 (19.23)	31 (15.58)	48 (14.46)	184 (17.08)	0.156^†^
Culprit lesion					
Left main disease	45 (8.24)	11 (5.53)	19 (5.72)	75 (6.96)	0.247^†^
Left anterior descending artery	282 (51.65)	106 (53.27)	169 (50.90)	557 (51.72)	0.869^†^
Right coronary artery	213 (39.01)	79 (39.70)	136 (40.96)	428 (39.74)	0.848^†^
Left circumflex artery	51 (9.34)	24 (12.06)	27 (8.13)	102 (9.47)	0.323^†^
Initial TIMI score					0.039^†^
0–1	372 (68.13)	138 (69.35)	201 (60.54)	711 (66.02)	
2–3	174 (31.87)	61 (30.65)	131 (39.65)	366 (33.98)	
Final TIMI score					0.561^†^
0–1	8 (1.47)	3 (1.51)	8 (2.41)	19 (1.76)	
2–3	538 (98.53)	196 (98.49)	324 (97.59)	1,058 (98.24)	
Medications at discharge					
Aspirin	542 (99.27)	199 (100)	332 (100)	1,073 (99.63)	0.313^†^
Adenosine diphosphate receptor inhibitors					<0.001^†^
None	8 (1.63)	2 (1.05)	4 (1.29)	14 (1.41)	
Clopidogrel	370 (75.20)	117 (61.58)	169 (54.69)	656 (66.20)	
Ticagrelor	112 (22.76)	70 (36.84)	122 (39.48)	304 (30.68)	
Prasugrel	2 (0.41)	1 (0.53)	14 (4.53)	17 (1.72)	
Statin					0.018^‡^
High-intensity atorvastatin	416 (87.03)	171 (92.93)	258 (85.43)	845 (87.66)	
High-intensity rosuvastatin	4 (0.84)	0 (0.00)	8 (2.65)	12 (1.24)	
Medium-intensity simvastatin	51 (10.67)	11 (5.98)	26 (8.61)	88 (9.13)	
Medium-intensity atorvastatin	6 (1.26)	1 (0.54)	6 (1.99)	13 (1.35)	
Medium-intensity rosuvastatin	0 (0.00)	0 (0.00)	2 (0.66)	2 (0.21)	
Medium-intensity pitavastatin	1 (0.21)	1 (0.54)	0 (0.00)	2 (0.21)	
Low-intensity simvastatin	0 (0.00)	0 (0.00)	2 (0.66)	2 (0.21)	
Beta antagonists					0.001^†^
None	275 (55.89)	122 (64.21)	147 (47.57)	544 (54.89)	
Carvedilol	154 (31.30)	53 (27.89)	120 (38.83)	327 (33.00)	
Metoprolol	51 (10.37)	12 (6.32)	24 (7.77)	87 (8.78)	
Other	12 (2.44)	3 (1.58)	18 (5.83)	33 (3.33)	
Angiotensin-converting enzyme inhibitors					0.821^†^
None	265 (53.86)	100 (52.63)	155 (50.16)	520 (52.47)	
Enalapril	219 (44.51)	88 (46.32)	150 (48.54)	457 (46.12)	
Other	8 (1.63)	2 (1.05)	4 (1.29)	14 (1.41)	

CS, Civil Service Reimbursement; SS, Social Security System; UC, Universal Health Coverage.

^†^ Chi-squared test.

^‡^ Fisher-exact test.

**Figure 2:**
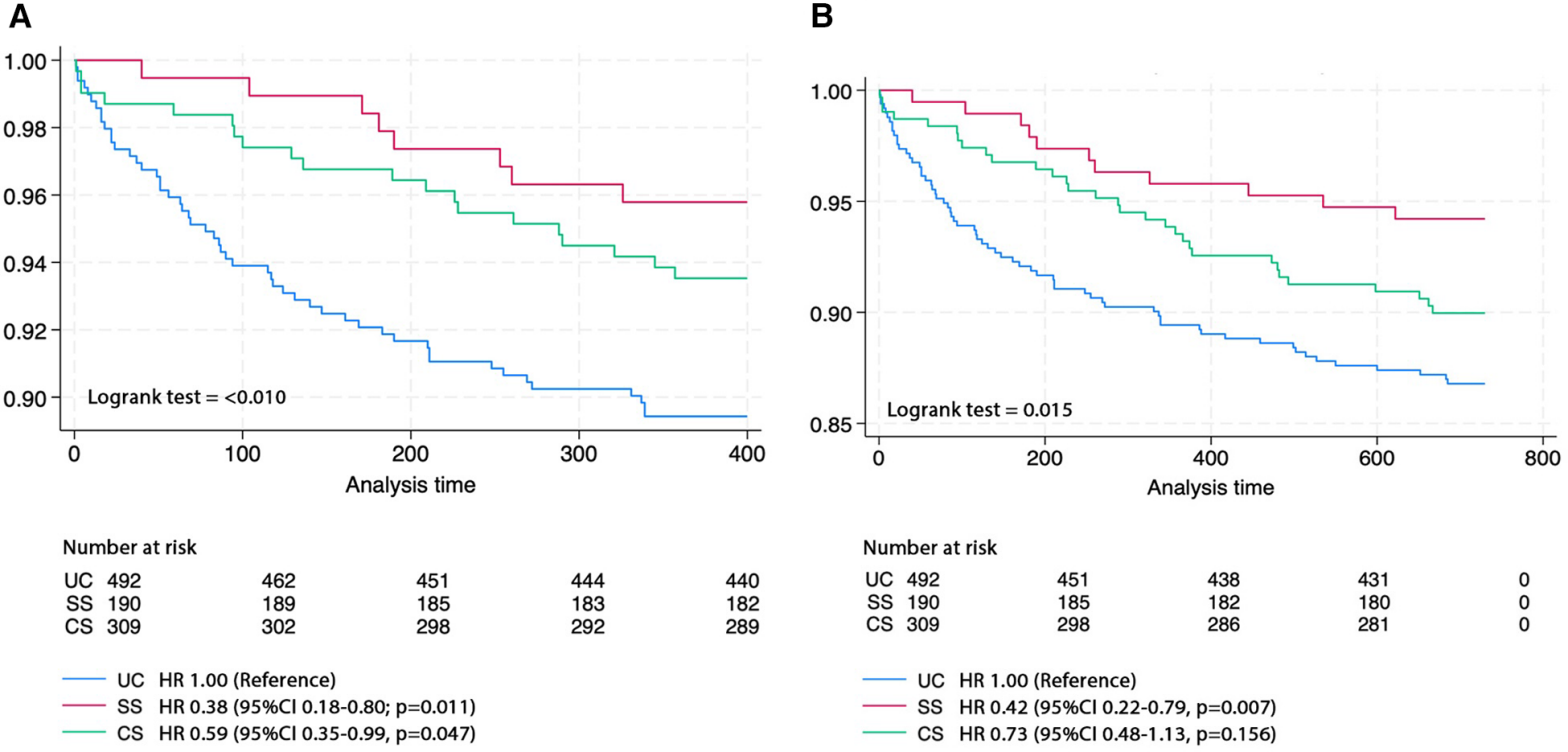
Kaplan-Meier curve of (**A**) one- and (**B**) two-year survival rates across the public health insurance schemes.

One-year follow-up observations, detailed in Table 5, indicated the CS group had the highest follow-up (75.00%) and readmission rate within one year (44.34%), primarily for staged PCI. Mortality rate at one- and two-years post-discharge revealed the highest death rates in the UC group, with the SS group recording the lowest figures. Kaplan–Meier curve was used to evaluate one- and two-year mortality rates across the public health insurance schemes (Figure 2) which revealed significant differences, with both the SS and CS groups demonstrating notably lower mortality rates at one-year compared to the UC group (*p* = 0.0083 and *p* = 0.0446, respectively). Also, the SS group maintained a significantly lower mortality rate at two-years compared to the UC group (*p* = 0.0055).

**Table 5:** Follow-up and rehospitalization of STEMI treated with pPCI.

Variables, *n* (%)	UC (*N* = 546)	SS (*N* = 199)	CS (*N* = 332)	Total patients (*N* = 1,077)	*p*-value^†^
Follow-up in KCMH	223 (40.84)	87 (43.72)	249 (75.00)	559 (51.90)	<0.001
Rehospitalization in KCMH	132 (26.83)	52 (27.37)	137 (44.34)	321 (32.39)	<0.001
Number of Rehospitalization in KCMH					0.397
1	93 (76.23)	38 (79.17)	89 (73.55)	220 (75.60)	
2	16 (13.11)	9 (18.75)	20 (16.53)	45 (15.46)	
>2	13 (10.66)	1 (2.08)	12 (9.92)	26 (8.93)	
Causes of Rehospitalization in KCMH					
Staged PCI	84 (63.64)	43 (82.69)	83 (60.58)	210 (65.42)	0.015
Heart failure	12 (9.09)	1 (1.92)	14 (10.22)	27 (8.41)	0.174
Ischemic heart diseases	9 (6.82)	5 (9.62)	5 (3.65)	19 (5.92)	0.255
One-year mortality in all patients	52 (10.57)	8 (4.21)	20 (6.47)	80 (8.07)	0.011
Two-year mortality in all patients	65 (13.21)	11 (5.79)	31 (10.03)	107 (10.80)	0.017

CS, Civil Service Reimbursement; KCMH, King Chulalongkorn Memorial Hospital; PCI, Percutaneous coronary intervention; SS, Social Security System; UC, Universal Health Coverage.

^†^ Chi-squared test.

Utilizing a multiple Cox regression model (Table 6), several predictors are associated with an increased risk of mortality within one-year post-discharge, including age equal to or more than 65 years, prior coronary artery diseases, Killip class 3–4, multivessel disease, occlusion at the left anterior descending artery, in-hospital atrial fibrillation/flutter and in-hospital pericardial effusion. Moreover, patients covered by the CS insurance and received pre-discharge angiotensin-converting enzyme inhibitor administration demonstrated a decreased risk of one-year post-discharge mortality.

**Table 6:** Cox proportional hazard multivariate analysis of predictors of one-year post-discharge mortality after pPCI.

Variables	Hazard ratio	95% Confidence interval	*p*-value
Universal Health Coverage	1.00	Reference	Reference
Social Security System	0.61	0.27–1.39	0.239
Civil Service Reimbursement	0.50	0.29–0.86	0.012
Age ≥ 65 years old	2.02	1.22–3.34	0.006
Prior coronary artery disease	2.02	1.14–3.56	0.016
Angiotensin-converting enzyme inhibitor	0.49	0.29–0.85	0.011
Killip class 3–4	2.17	1.31–3.60	0.003
Left anterior descending artery	2.57	1.56–4.23	<0.001
Multivessel disease	1.76	1.02–3.01	0.041
Pericardial effusion	13.34	3.75–47.51	<0.001
Atrial fibrillation/flutter	2.28	1.30–4.01	0.004

## 4 Discussion

This study represents the first national investigation into the correlation between public health insurance schemes in Thailand and the long-term mortality rates among STEMI patients receiving pPCI treatment. Our findings reveal that different health insurance schemes have a statistically significant impact on mortality rates. Specifically, the SS group demonstrated the lowest one-year mortality rate at 4.21%, followed by the CS group at 6.47%. Furthermore, a longitudinal analysis extending to two years supported that the SS cohort consistently showed the lowest mortality rate. Additionally, international studies underscore that the entitlement to treatment profoundly influences both the management and outcomes of patients (9–12).

Upon examining the basic characteristics, the SS group was found to have the youngest average age. We observed that patients aged 65 years or older had nearly twice the risk of mortality within one year, which aligns with previous research indicating that age is a significant predictor of mortality (13, 14). When comparing the basic characteristics of patients in other studies, the SS group was found to be younger than the sample groups in other studies (3, 9–11). In terms of comorbidities, the SS patient group had the lowest number of diabetes mellitus, which is a predictive factor for short-term and long-term mortality (15, 16). Regarding smoking history, a higher proportion of SS patients were smokers. This finding is consistent with observations in smokers with acute myocardial infarction where the paradoxical protective effect of smoking may be attributed to smoking being a confounding variable (17). When assessing the severity at hospital admission using the Killip classification, other studies have indicated that higher scores are associated with an increased risk of mortality in both the short-term and long-term (18), which is consistent with our findings. The SS patient group had a lower proportion of high Killip class.

Upon further examination of laboratory blood values, the SS patient group exhibited the lowest peak creatinine levels. Previous research has established that elevated creatinine levels are indicative of an increased risk of mortality (13). Our study similarly found that patients with acute kidney injury faced a heightened risk of in-hospital mortality. Additionally, the highest levels of LDL in the SS group were observed in comparison to other groups. This phenomenon can be elucidated by the LDL paradox, where it has been documented those patients with elevated LDL levels during myocardial ischemia experienced reduced mortality and morbidity, likely attributed to younger age and fewer comorbidities (19).

Shortening the treatment delay time significantly influences mortality rates (20). In our research, the SS patient group exhibited the shortest diagnosis-to-wire time, yet, regrettably, this time frame did not appear as a protective factor. Nonetheless, we encourage that reducing pre-hospital time presents a critical opportunity to enhance treatment outcomes and should be prioritized for additional attention and resource allocation (21).

In terms of procedures, SS patients received DES more frequently than BMS, with DES being the preferred choice of stent used in the procedure (22). This results in superior outcomes in terms of reducing the rate of re-occlusion of the stents (6). The final TIMI score does not differ among the three groups, along with the mortality rate in the catheterization room being similar.

In the comparison between the Civil Servant (CS) and Universal Coverage (UC) groups, both exhibit similar baseline characteristics and receive similar treatment during hospitalization. However, the CS group demonstrates a lower one-year mortality rate. Being covered under the CS scheme serves as a protective factor against one-year mortality. This can be attributed to the extensive coverage offered by the CS insurance, which grants patients access to treatment at any public hospital, in contrast to the UC and SS schemes that limit treatment to registered hospitals only. Additionally, patients under the CS scheme have access to a wider range of medications, including those not on the National List of Essential Medicine (NLEM), original drugs, and those with higher prices. Notably, Clopidogrel, an ADP inhibitor, is available to all CS patients without additional cost (8). Furthermore, CS patients are more likely to receive the predischarge non-Clopidogrel medications, such as Ticagrelor or Prasugrel, which may result in a lower recurrence of myocardial infarctions in CS group.

When reviewing the medications provided at discharge, it shows that patients in the CS group are more likely to receive angiotensin converting enzyme inhibitors and beta-blockers compared to other health insurance groups. Renin-angiotensin system inhibitors and beta-antagonists are associated with enhanced outcomes in both short-term and long-term cardiovascular diseases (5, 6). The administration of angiotensin-converting enzyme inhibitors to the CS patients prior to discharge, which is a protective factor, may lead to a lower mortality rate among the CS patients compared to others. However, nearly half of the patients still did not receive beta-antagonists or angiotensin-converting enzyme inhibitors, a continuing issue from previous research (3). Improving the administration of these medications could present an opportunity to reduce morbidity and mortality in STEMI patients.

Moreover, follow-up data indicates that the CS group have the highest rate of one-year treatment adherence. This may be crucial for secondary prevention of STEMI patients. Additionally, CS group have a higher rate of hospitalizations, with more than half of these patients scheduled for staged PCI. The CS group shows a significantly higher rate of hospitalization compared to other groups. However, the greater number of CS patients who undergo follow-up at KCMH ensures that the rehospitalization data pertaining to this group is more precise and accurate. Nonetheless, future studies should aim to collect data on patient follow-ups from all hospitals where treatment is received.

In addition, the reason the CS group has a higher rate of hospitalizations may possibly be due to the direct effects of hospital payments and patient demand for services. The CS scheme reimburses hospitals based on the actual costs of services rendered. This contrasts with other systems that use capitation or Diagnosis-Related Groups (DRG), which may result in hospitals receiving less than their expenditures (8). It is interesting that despite the higher incidence of in-hospital complications among UC group, they experience a less hospitalization duration compared to the CS group. This may be explained by the fact that CS patients tend to request more services since they are aware that the hospital is entitled to full reimbursement of expenses by the government. Although the SS group cohort showed lower in-hospital and one-year mortality rates, upon conducting a multivariable analysis, it was determined that this factor did not serve as a protective variable against mortality. This may be attributed to the younger age demographic, lower Killip class, and lower in-hospital complications.

Our research indicates that healthcare schemes play a significant role in influencing mortality rates among patients. Health equity implies the equitable and appropriate access to high-quality health services for all individuals. Also, distributing equity across different regions is crucial (23). The government aims to evolve the health insurance system to augment quality and extend coverage nationwide, particularly through the UC scheme, which covers most of the population (24). The assessment of PCI treatment in UC patients has been increasing, resulting in a noticeable reduction in mortality rates (25).

### 4.1 Limitations

Cost-effectiveness was not presented in this study as some patients were transferred out of the hospital before recovery, making it difficult to track expenses. In searching for mortality data in the civil registration system, information on the cause of death was not provided. The study was conducted on data from only one tertiary care hospital, resulting in limited patient data distribution which may not accurately represent the treatment outcomes in general. Therefore, further prospective research is required to gather data on a national level.

## 5 Conclusion

We found that the one-year mortality rate of patients with STEMI treated with pPCI in Thailand tends to be lower. Patients using the SS scheme had the lowest one-year and two-year mortality rates, followed by patients with the CS scheme, which is consistent with the in-hospital mortality rate. The CS schemes and the pre-discharge administration of angiotensin-converting enzyme inhibitors independently serve as protective factors against one-year mortality. In contrast, advanced age, a Killip class of 3–4 at hospital admission, multivessel disease, occlusion in the left anterior descending artery, and the presence of atrial fibrillation/flutter and pericardial effusion independently elevate the risk of one-year mortality. The improvement of the UC scheme may reduce morbidity and mortality in Thailand.

## Data Availability

The raw data supporting the conclusions of this article will be made available by the authors, without undue reservation.
