# Supplementary material for: Mortality rate of percutaneous coronary interventions in ST-segment elevation myocardial infarction patients under the public health insurance schemes of Thailand
Source: Front Cardiovasc Med. 2024 Oct 4;11:1397015. doi: 10.3389/fcvm.2024.1397015 (PMC11486745; doi:10.3389/fcvm.2024.1397015)
Supplement: Supplementary file 1 [file Table1.docx]

Supplementary Material

# Supplementary Tables

| Supplementary Table 1. Univariate logistic analysis of predictors of in-hospital mortality after pPCI | | | |
| --- | --- | --- | --- |
| Variables | Odds ratio | 95% Confidence interval | p-value |
| Healthcare |  |  |  |
| Universal Health Coverage | 1.00 | Referent | Referent |
| Social Security System | 0.43 | 0.21 – 0.89 | 0.023 |
| Civil Service Reimbursement | 0.68 | 0.41 – 1.13 | 0.134 |
| Female | 2.05 | 1.3 – 3.23 | 0.002 |
| Age ≥ 65 | 2.34 | 1.5 – 3.64 | <0.001 |
| Underlying diseases |  |  |  |
| Diabetic mellitus | 1.79 | 1.15 – 2.79 | 0.01 |
| Hypertension | 1.88 | 1.14 – 3.10 | 0.014 |
| Cerebrovascular diseases | 2.64 | 1.42 – 4.92 | 0.002 |
| Chronic kidney diseases | 3.40 | 1.97 – 5.90 | <0.001 |
| Previous Myocardial Infarction | 2.06 | 1.15 – 3.68 | 0.015 |
| Complications |  |  |  |
| Killip 3 – 4 | 19.92 | 11.53 – 34.41 | <0.001 |
| Arrest at admission | 11.64 | 6.89 – 19.67 | <0.001 |
| Pericardial effusion | 16.84 | 5.70 – 49.75 | <0.001 |
| Bradycardia | 3.08 | 1.97 – 4.81 | <0.001 |
| Atrial fibrillation/flutter | 7.48 | 4.62 – 12.11 | <0.001 |
| Complete heart block | 3.65 | 2.05 – 6.49 | <0.001 |
| Ventricular tachycardia | 12.56 | 6.84 – 23.05 | <0.001 |
| Infection | 7.56 | 4.71 – 12.13 | <0.001 |
| Acute kidney failure | 12.11 | 7.53 – 19.50 | <0.001 |
| Ischemic hepatitis | 6.49 | 3.66 – 11.50 | <0.001 |
| Lesion |  |  |  |
| Multivessels disease | 2.90 | 1.66 – 5.06 | <0.001 |
| Left main artery | 3.61 | 1.97 – 6.60 | <0.001 |
| Stents |  |  |  |
| Drug-eluting stent | 0.46 | 0.29 – 0.74 | 0.001 |
| Plain Old Balloon Angioplasty | 2.74 | 1.70 – 4.43 | <0.001 |

| Supplementary Table 2. Cox proportional hazard univariate analysis of predictors of one-year post-discharge mortality after pPCI | | | |
| --- | --- | --- | --- |
| Variables | Hazard ratio | 95% Confidence interval | p-value |
| Healthcare |  |  |  |
| Universal Health Coverage | 1.00 | Referent | Referent |
| Social Security System | 0.38 | 0.18 – 0.80 | 0.011 |
| Civil Service Reimbursement | 0.59 | 0.35 – 0.99 | 0.047 |
| Female | 1.83 | 1.16 – 2.88 | 0.009 |
| Age ≥ 65 | 2.46 | 1.59 – 3.82 | <0.001 |
| Underlying diseases |  |  |  |
| Hypertension | 2.27 | 1.34 – 3.84 | 0.002 |
| Cerebrovascular diseases | 2.27 | 1.20 – 4.29 | 0.012 |
| Chronic kidney disease | 2.95 | 1.70 – 5.09 | <0.001 |
| Previous myocardial infarction | 2.52 | 1.47 – 4.30 | 0.001 |
| Complications |  |  |  |
| Killip 3 – 4 | 3.21 | 2.04 – 5.07 | <0.001 |
| Pericardiac effusion | 8.81 | 2.78 – 27.96 | <0.001 |
| Atrial fibrillation/flutter | 3.84 | 2.32 – 6.38 | <0.001 |
| Infection | 3.29 | 1.98 – 5.45 | <0.001 |
| Acute kidney injury | 4.05 | 2.54 – 6.47 | <0.001 |
| Home medication |  |  |  |
| Adenosine diphosphate receptor inhibitors | | | |
| None | 1.00 | Referent | Referent |
| Clopidogrel | 0.31 | 0.11 – 0.84 | 0.022 |
| Ticagrelor | 0.22 | 0.07 – 0.64 | 0.005 |
| Angiotensin-converting enzyme inhibitors | 0.33 | 0.20 – 0.56 | <0.001 |
| Statin |  |  |  |
| Moderate-intensity Atorvastatin | 1.00 | Referent | Referent |
| High-intensity Atorvastatin | 0.28 | 0.09 – 0.90 | 0.033 |
| Lesion |  |  |  |
| Multivessels disease | 1.68 | 1.03 – 2.73 | 0.036 |
| Left anterior descending artery | 1.58 | 1.01 – 2.49 | 0.047 |
| Stents |  |  |  |
| Drug-eluting stent | 0.61 | 0.37 – 0.99 | 0.044 |
| Plain Old Balloon Angioplasty | 1.80 | 1.08 – 2.98 | 0.023 |

| Supplementary Table 3. In-hospital medication | | | | | |
| --- | --- | --- | --- | --- | --- |
| Variables – n (%) | UC  (N= 546) | SS  (N= 199) | CS  (N= 332) | All patient  (N= 1077) | p-value |
| Pre-procedure coronary angiography | | | | | |
| Adenosine diphosphate receptor inhibitors | | | | | <0.001* |
| None | 1 (0.18) | 1 (0.50) | 4 (1.20) | 6 (0.56) |  |
| Clopidogrel | 425 (77.84) | 125 (62.81) | 225 (67.77) | 775 (71.96) |  |
| Ticagrelor | 119 (21.79) | 72 (36.18) | 95 (28.61) | 286 (26.56) |  |
| Prasugrel | 1 (0.18) | 1 (0.50) | 8 (2.41) | 10 (0.93) |  |
| In-hospital Medications | | |  |  |  |
| Norepinephrine | 43 (7.89) | 7 (3.52) | 24 (7.23) | 74 (6.88) | 0.108^†^ |
| Adrenaline | 85 (15.60) | 22 (11.06) | 39 (11.75) | 146 (13.57) | 0.141^†^ |
| Dopamine | 148 (27.11) | 42 (21.11) | 84 (25.30) | 274 (25.44) | 0.250^†^ |
| Dobutamine | 25 (4.59) | 7 (3.52) | 17 (5.12) | 49 (4.55) | 0.691^†^ |
| Atropine | 110 (20.18) | 49 (24.62) | 74 (22.29) | 233 (21.65) | 0.405^†^ |
| Digoxin | 14 (2.56) | 0 (0.00) | 7 (6.5) | 21 (1.95) | 0.079^†^ |
| Amiodarone | 77 (14.10) | 18 (9.05) | 42 (12.65) | 137 (12.72) | 0.186^†^ |
| Furosemide | 221 (40.48) | 46 (23.12) | 129 (38.86) | 396 (36.77) | <0.001^†^ |
| Isosorbide dinitrate | 303 (55.49) | 121 (60.80) | 211 (63.55) | 635 (58.96) | 0.053^†^ |
| Nitroglycerin | 240 (43.96) | 86 (43.22) | 141 (42.47) | 467 (43.36) | 0.910^†^ |
| Heparin | 536 (98.17) | 196 (98.49) | 322 (96.99) | 1054 (97.86) | 0.399^†^ |
| Glycoprotein IIb/IIIa | 410 (75.09) | 150 (75.38) | 247 (74.40) | 807 (74.93) | 0.961^†^ |
| CS, Civil Service Reimbursement; SS, Social Security System; UC, Universal Health Coverage  * Fisher-exact test  † Chi-squared test | | | | | |

# AI use by authors

Thai Healthcare Disparities Study

User

American spelling and grammar check this abstract: Background: ST-elevation myocardial infarction (STEMI) presents a critical public health challenge internationally, including in Thailand. Currently, standard therapeutic interventions, particularly primary percutaneous coronary interventions (pPCI), are administered to Thai citizens. However, selected healthcare systems restrict access to specific pharmaceuticals and medical devices, causing inequalities in the quality of medical care amongst different healthcare systems. Such inequalities have the potential to significantly impact the quality of life of patients, particularly in the long term.

Objective: The primary objective is to compare mortality rates within one year of STEMI patients after treatment with pPCI among the public health insurance schemes of Thailand. The secondary objectives are to study prognostic determinants for one-year mortality rates among patients diagnosed with STEMI who have undergone pPCI.

Methodology: This study is a single-centre retrospective analysis of patients with STEMI treated with pPCI. It involves patients utilising various state health insurance schemes in Thailand from 1 January 2010 to 31 December 2020. Data collection occurred through the hospital's computerised management system and the registration administration office of the Department of Provincial Administration. The data analysis employed the Kaplan-Meier survival analysis, log-rank test, and Cox proportional hazards regression modelling method.

Results: The study involved 1,077 patients, categorised into three groups based on their state health insurance: Universal Health Coverage (UC) (546 patients, 50.7%), Social Security System (SS) (199 patients, 18.5%), and Civil Service Reimbursement (CS) (332 patients, 62.6%). The one-year mortality rates in these groups were 10.57%, 4.21%, and 6.47%, respectively (p= 0.010). In the unadjusted model, the SS group showed a lower risk of one-year mortality (HR 0.38, 95% CI 0.18-0.80, p=0.011), and the CS group also demonstrated a lower risk (HR 0.59, 95% CI 0.35-0.99, p=0.047) compared to the UC group. In the adjusted model, only the CS group significantly reduced the risk of one-year mortality. Other factors that affected one-year mortality were age ≥65 years, a Killip score of 3-4, pre-discharge prescription of angiotensin-converting enzyme inhibitors, occlusion in the left anterior descending artery, and pericardial effusion.

Conclusion: Healthcare schemes play a significant role in influencing one-year mortality rates among STEMI patients treated with pPCI. The use of civil service reimbursement is a protective factor for the one-year mortality rate in STEMI patients. This information would be crucial for developing strategies and programs to aid healthcare policymakers at both regional and international levels in reducing morbidity and mortality.

ChatGPT

Here is the abstract with American spelling and grammar corrections:

Background: ST-elevation myocardial infarction (STEMI) presents a critical public health challenge internationally, including in Thailand. Currently, standard therapeutic interventions, particularly primary percutaneous coronary interventions (pPCI), are administered to Thai citizens. However, selected healthcare systems restrict access to specific pharmaceuticals and medical devices, causing inequalities in the quality of medical care among different healthcare systems. Such inequalities have the potential to significantly impact the quality of life of patients, particularly in the long term.

Objective: The primary objective is to compare mortality rates within one year of STEMI patients after treatment with pPCI among the public health insurance schemes of Thailand. The secondary objectives are to study prognostic determinants for one-year mortality rates among patients diagnosed with STEMI who have undergone pPCI.

Methodology: This study is a single-center retrospective analysis of patients with STEMI treated with pPCI. It involves patients utilizing various state health insurance schemes in Thailand from January 1, 2010, to December 31, 2020. Data collection occurred through the hospital's computerized management system and the registration administration office of the Department of Provincial Administration. The data analysis employed the Kaplan-Meier survival analysis, log-rank test, and Cox proportional hazards regression modeling method.

Results: The study involved 1,077 patients, categorized into three groups based on their state health insurance: Universal Health Coverage (UC) (546 patients, 50.7%), Social Security System (SS) (199 patients, 18.5%), and Civil Service Reimbursement (CS) (332 patients, 62.6%). The one-year mortality rates in these groups were 10.57%, 4.21%, and 6.47%, respectively (p = 0.010). In the unadjusted model, the SS group showed a lower risk of one-year mortality (HR 0.38, 95% CI 0.18-0.80, p = 0.011), and the CS group also demonstrated a lower risk (HR 0.59, 95% CI 0.35-0.99, p = 0.047) compared to the UC group. In the adjusted model, only the CS group significantly reduced the risk of one-year mortality. Other factors that affected one-year mortality were age ≥65 years, a Killip score of 3-4, pre-discharge prescription of angiotensin-converting enzyme inhibitors, occlusion in the left anterior descending artery, and pericardial effusion.

Conclusion: Healthcare schemes play a significant role in influencing one-year mortality rates among STEMI patients treated with pPCI. The use of civil service reimbursement is a protective factor for the one-year mortality rate in STEMI patients. This information would be crucial for developing strategies and programs to aid healthcare policymakers at both regional and international levels in reducing morbidity and mortality.

User

ST-elevation myocardial infarction (STEMI) presents a critical public health challenge internationally, including in Thailand. In Thailand, selected healthcare systems restrict access to specific pharmaceuticals and medical devices, causing inequalities in the quality of medical care among different healthcare systems. Such inequalities have the potential to significantly impact the quality of life of patients, particularly in the long term. Objective: The primary objective is to compare mortality rates within one year of STEMI patients after treatment with pPCI among the public health insurance schemes of Thailand.

ChatGPT

Here is the revised section with American spelling and grammar corrections:

ST-elevation myocardial infarction (STEMI) presents a critical public health challenge internationally, including in Thailand. In Thailand, selected healthcare systems restrict access to specific pharmaceuticals and medical devices, causing inequalities in the quality of medical care among different healthcare systems. Such inequalities have the potential to significantly impact the quality of life of patients, particularly in the long term.

Objective: The primary objective is to compare mortality rates within one year of STEMI patients after treatment with primary percutaneous coronary intervention (pPCI) among the public health insurance schemes of Thailand.

User

Within Thailand, certain healthcare systems impose restrictions on access to specific pharmaceuticals and medical devices, leading to disparities in the quality of medical care across different healthcare systems.

ChatGPT

Here's a slightly revised version for clarity:

Within Thailand, certain healthcare systems impose restrictions on access to specific pharmaceuticals and medical devices, resulting in disparities in the quality of medical care across various healthcare systems.

User

In Thailand, certain healthcare systems impose restrictions on access to specific pharmaceuticals and medical devices, leading to disparities in the quality of medical care across different healthcare systems.

ChatGPT

Your sentence is already clear and formal. If you're looking for a slight variation, you could consider:

"In Thailand, specific healthcare systems enforce restrictions on the availability of particular pharmaceuticals and medical devices, resulting in disparities in the quality of medical care among different healthcare systems."

User

In Thailand, certain healthcare systems are restricted to access to specific pharmaceuticals and medical devices, causing inequalities in the quality of medical care among different healthcare systems.

ChatGPT

Your sentence is clear, but for improved clarity, you might consider:

"In Thailand, access to specific pharmaceuticals and medical devices is restricted within certain healthcare systems, leading to inequalities in the quality of medical care among different healthcare systems."

User

In Thailand, access to specific pharmaceuticals and medical devices for ST-elevation myocardial infarction (STEMI) patients presents is restricted within certain healthcare systems, leading to inequalities in the quality of medical care among different healthcare systems.

ChatGPT

Your sentence is clear, but there seems to be a small typo. Here's a corrected version:

"In Thailand, access to specific pharmaceuticals and medical devices for ST-elevation myocardial infarction (STEMI) patients is restricted within certain healthcare systems, leading to inequalities in the quality of medical care among different healthcare systems."

User

American spelling and grammar check this paragraphs: ST-segment elevation myocardial infarction (STEMI) contributes a major public health challenge worldwide, including Thailand(1). The prevalence of STEMI within the nation displays an ascending trend, leading to prominent rates of morbidity and mortality(2-4). Recommended by international guidelines, primary percutaneous coronary intervention (pPCI) is currently a preferred therapeutic intervention for STEMI patients, when performed by experts within an optimal timeframe(5, 6). Compared with tradition intervention, thrombolytic therapy, pPCI demonstrates superior efficacy which significantly reduces short-term and long-term mortality rates, the incidence of recurrent arterial occlusions, the risk of cerebrovascular events, and the occurrence of abnormal bleeding(7).

In Thailand, majority of healthcare services is predominantly provided by the government sector. Healthcare services in Thailand are divided into three distinct systems: Universal Health Coverage (UC), Social Security System (SS), and Civil Service Reimbursement (CS), which cover 75%, 16%, and 9% of the population, respectively. The UC objective is to provide healthcare services without incurring personal financial costs for Thai citizens. SS is intended for private sector employees, providing a safety net for life sustenance through contributions from employers, employees, and the state. CS is a privilege for active and retired civil servants, representing one of the welfare benefits provided by the government(8).

Each system has its own conceptual underpinnings, leading to inequalities in public health service access among the three types of health coverages. This results in discrepancies in access to medications, and variations in the quality of treatments leading to a potential to significantly impact the quality of life of patients, both in the short and long-term. Therefore, the authors sought to determine the difference in patients’ characteristics, procedure variations, in-hospital outcomes, and long-term outcomes among the healthcare systems. This information would be crucial for developing strategies and programs to aid healthcare policymakers at both regional and international levels in reducing morbidity and mortality.

User

Study design

This study is a descriptive, retrospective research on patients diagnosed with ST-elevation myocardial infarction (STEMI), who received primary percutaneous coronary intervention (pPCI), at the King Chulalongkorn Memorial Hospital, a tertiary referral center. The data for this study was complied from the King Chulalongkorn Memorial Hospital's database management system, utilizing International Classification of Diseases, Tenth Revision, Clinical Modification (ICD-10-CM) codes I21.0 to I21.4, and the Ninth Revision, Clinical Modification (ICD-9-CM) codes 00.40 to 0048, 00.6, and 36.06 to 36.07. The period under review in this study extends from January 1, 2010, through December 31, 2020.

We identified and recorded the total number of patients who met the pre-defined inclusion criteria: Patients who received the primary PCI strategy as the initial treatment, and whose age was equal to or greater than 18 years. We excluded patients with missing data on national identification number, health insurance coverage under the Thai national health insurance scheme, or no history on civil registration system, Patients who received the pharmacoinvasive strategy, and Patients who underwent coronary artery bypass grafting (CABG) surgery. Patients were followed for mortality from the time of procedure initiation. However, the analysis exclusively included deaths that occurred post-procedure. Demographic and clinical information, including laboratory test results and procedural data, were retrospectively collected by well-trained critical care nurses and cardiologists, and documented from both outpatient and inpatient database records. Routine follow-up data collection included hospital visits and assessments at 12 months via file review for specific clinical measures, patient-reported outcomes, or hospitalizations.

For verifying the timing of deaths, our study was granted access to the civil registration system through the Bureau of Registration Administration (BORA) of Thailand. We performed a matching process using the patient's national identification number and date of birth against entries in the civil registration system. A match was confirmed when an entry in the Index corresponded exactly in patient's national identification number and date of birth with a patient recorded in our study registry.

This study was conducted in adherence to the principles of Good Clinical Practice, adhering to all relevant Thai legislation and data protection regulations. No individual specific consent forms for the study were obtained. The protocol was reviewed and approved by an ethics committee.

Statistical analysis

The primary objective is to compare mortality rates within one year of STEMI patients after treatment of pPCI among the public health insurance schemes of Thailand. The secondary objectives are to study prognostic determinants for both in-hospital and one-year mortality rates among patients diagnosed with STEMI who have undergone pPCI at King Chulalongkorn Memorial Hospital.

Categorical variables are presented as frequency and proportion using the Pearson Chi-square test or Fisher’s exact test, while continuous variables are expressed through mean, standard deviations (SD), medians, or interquartile ranges (IQR) analyzed with the student's t-test, one-way analysis of variance (ANOVA), or Kruskal-Wallis test. We study aims to conduct a comparative analysis of one-year mortality rates across different health care entitlements in Thailand, including the Universal Health Coverage, Social Security System, and Civil Service Reimbursement. To assess differences in mortality outcomes among these groups, Kaplan-Meier survival analysis and log-rank testing methodologies are utilized. Multiple logistic regression analyses were executed to determine the predictors of in-hospital outcomes, while multiple cox regression model to compute crude hazard ratios are utilized to examine the individual relation between each predictor and death during one-year follow-up. Variables selected for inclusion in the final multivariate models were those demonstrating a significance level of less than 0.05 in the univariate analyses, unless otherwise stated. All statistical tests were conducted as two-sided, with the threshold for statistical significance established at a P-value of less than 0.05. All statistical analyses will be performed using STATA version 17 for MacOS (StataCorp. 2021. Stata Statistical Software: Release 17. College Station, TX: StataCorp LP).

ChatGPT

Here are the paragraphs with American spelling and grammar corrections:

**Study design**

This study is a descriptive, retrospective research on patients diagnosed with ST-elevation myocardial infarction (STEMI), who received primary percutaneous coronary intervention (pPCI), at the King Chulalongkorn Memorial Hospital, a tertiary referral center. The data for this study was compiled from the King Chulalongkorn Memorial Hospital's database management system, utilizing International Classification of Diseases, Tenth Revision, Clinical Modification (ICD-10-CM) codes I21.0 to I21.4, and the Ninth Revision, Clinical Modification (ICD-9-CM) codes 00.40 to 0048, 00.6, and 36.06 to 36.07. The period under review in this study extends from January 1, 2010, through December 31, 2020.

We identified and recorded the total number of patients who met the pre-defined inclusion criteria: Patients who received the primary PCI strategy as the initial treatment, and whose age was equal to or greater than 18 years. We excluded patients with missing data on national identification number, health insurance coverage under the Thai national health insurance scheme, or no history in the civil registration system, patients who received the pharmacoinvasive strategy, and patients who underwent coronary artery bypass grafting (CABG) surgery. Patients were followed for mortality from the time of procedure initiation. However, the analysis exclusively included deaths that occurred post-procedure. Demographic and clinical information, including laboratory test results and procedural data, were retrospectively collected by well-trained critical care nurses and cardiologists, and documented from both outpatient and inpatient database records. Routine follow-up data collection included hospital visits and assessments at 12 months via file review for specific clinical measures, patient-reported outcomes, or hospitalizations.

For verifying the timing of deaths, our study was granted access to the civil registration system through the Bureau of Registration Administration (BORA) of Thailand. We performed a matching process using the patient's national identification number and date of birth against entries in the civil registration system. A match was confirmed when an entry in the Index corresponded exactly to a patient's national identification number and date of birth with a patient recorded in our study registry.

This study was conducted in adherence to the principles of Good Clinical Practice, adhering to all relevant Thai legislation and data protection regulations. No individual specific consent forms for the study were obtained. The protocol was reviewed and approved by an ethics committee.

**Statistical analysis**

The primary objective is to compare mortality rates within one year of STEMI patients after treatment of pPCI among the public health insurance schemes of Thailand. The secondary objectives are to study prognostic determinants for both in-hospital and one-year mortality rates among patients diagnosed with STEMI who have undergone pPCI at King Chulalongkorn Memorial Hospital.

Categorical variables are presented as frequency and proportion using the Pearson Chi-square test or Fisher’s exact test, while continuous variables are expressed through mean, standard deviations (SD), medians, or interquartile ranges (IQR) analyzed with the student's t-test, one-way analysis of variance (ANOVA), or Kruskal-Wallis test. The study aims to conduct a comparative analysis of one-year mortality rates across different healthcare entitlements in Thailand, including the Universal Health Coverage, Social Security System, and Civil Service Reimbursement. To assess differences in mortality outcomes among these groups, Kaplan-Meier survival analysis and log-rank testing methodologies are utilized. Multiple logistic regression analyses were executed to determine the predictors of in-hospital outcomes, while multiple Cox regression models to compute crude hazard ratios are utilized to examine the individual relation between each predictor and death during one-year follow-up. Variables selected for inclusion in the final multivariate models were those demonstrating a significance level of less than 0.05 in the univariate analyses, unless otherwise stated. All statistical tests were conducted as two-sided, with the threshold for statistical significance established at a P-value of less than 0.05. All statistical analyses will be performed using STATA version 18 for MacOS (StataCorp. 2023. Stata Statistical Software: Release 18. College Station, TX: StataCorp LP).

User

In this study, we examined data from 1,776 STEMI patients who underwent primary PCI from January 1, 2010, to December 31, 2020. Following the application of exclusion criteria, which led to the removal of 699 patients as depicted in Figure 1, our analysis focused on the remaining 1,077 participants. These individuals were stratified into three distinct groups based on their enrollment in public health insurance schemes of Thailand: 546 patients in the Universal Health Coverage (UC) group (50.69%), 199 patients in the Social Security System (SS) group (18.48%), and 332 patients in the Civil Service Reimbursement (CS) group (30.83%). Baseline demographic and clinical characteristics, presented in Table 1, reveal that the SS cohort exhibited a higher male predominance (86.90%), the youngest mean age (51.62 years), and the most significant proportion of transferred patients (71.90%). Notably, this group also reported the highest proportion of Killip score I at hospital admission (79.90%). Laboratory findings showed the lowest mean serum creatinine levels in the SS group at 0.945 mg/dl and the lowest LDL levels in the CS group at 121 mg/dl.

Significant statistical differences between groups were observed in hospital complications (Table 2), including cardiac arrest with non-shockable rhythm, atrial fibrillation/atrial flutter, heart failure, acute kidney injury, and infections. Remarkably, these complications were least prevalent in the SS group, with in-hospital mortality also lowest in this cohort. Through multiple logistic regression analysis (Table 3), variables for in-hospital mortality were identified, including cardiac arrest upon admission and advanced Killip scores of 3-4. Additional factors included multivessel diseases and left main artery involvement, alongside complications such as pericardiac effusion, atrial fibrillation/flutter, ventricular tachycardia, and acute kidney failure.

In terms of cardiac catheterization procedures, as presented in Table 4, the SS group had the highest proportion of procedures completed within the to the Diagnosis-to-Wire time of less than 90 minutes (94.95%), and also achieved the highest proportion within <120 minutes (55.10%) drug-eluting stents (DES) appeared to be the predominant choice (78.92%), followed by plain old balloon angioplasty (POBA) and bare-metal stents (BMS) at 17.08% and 8.91%, respectively. The SS cohort led in DES utilization (85.43%), with the UC and CS groups behind at 80.95% and 71.69%, respectively.

One-year follow-up observations, detailed in Table 5, indicated the CS group had the highest follow-up (75.00%) and readmission rate within one year (44.34%), primarily for staged PCI. Mortality rate at one- and two-years post-discharge revealed the highest death rates in the UC group, with the SS group recording the lowest figures. Kaplan–Meier survival analysis was used to evaluate one- and two-year mortality rates across the public health insurance schemes (Figure 2) which revealed significant differences, with both the SS and CS groups demonstrating notably lower mortality rates at one-year compared to the UC group (p=0.0083 and p=0.0446, respectively). Also, the SS group maintained a significantly lower mortality rate at two-years compared to US group (p=0.0055).

Utilizing a multiple cox regression model (Table 6), several predictors are associated with an increased risk of mortality within one-year post-discharge, including age equal or more than 65 years, Killip scores 3-4, pre-discharge angiotensin-converting enzyme inhibitor administration, occlusion at the left anterior descending artery and in-hospital pericardial effusion. Moreover, patients covered by the CS insurance demonstrated a decreased risk of 1-year post-discharge mortality.

ChatGPT

Here are the paragraphs with American spelling and grammar corrections:

In this study, we examined data from 1,776 STEMI patients who underwent primary PCI from January 1, 2010, to December 31, 2020. Following the application of exclusion criteria, which led to the removal of 699 patients as depicted in Figure 1, our analysis focused on the remaining 1,077 participants. These individuals were stratified into three distinct groups based on their enrollment in public health insurance schemes of Thailand: 546 patients in the Universal Health Coverage (UC) group (50.69%), 199 patients in the Social Security System (SS) group (18.48%), and 332 patients in the Civil Service Reimbursement (CS) group (30.83%). Baseline demographic and clinical characteristics, presented in Table 1, reveal that the SS cohort exhibited a higher male predominance (86.90%), the youngest mean age (51.62 years), and the most significant proportion of transferred patients (71.90%). Notably, this group also reported the highest proportion of Killip score I at hospital admission (79.90%). Laboratory findings showed the lowest mean serum creatinine levels in the SS group at 0.945 mg/dl and the lowest LDL levels in the CS group at 121 mg/dl.

Significant statistical differences between groups were observed in hospital complications (Table 2), including cardiac arrest with non-shockable rhythm, atrial fibrillation/atrial flutter, heart failure, acute kidney injury, and infections. Remarkably, these complications were least prevalent in the SS group, with in-hospital mortality also lowest in this cohort. Through multiple logistic regression analysis (Table 3), variables for in-hospital mortality were identified, including cardiac arrest upon admission and advanced Killip scores of 3-4. Additional factors included multivessel diseases and left main artery involvement, alongside complications such as pericardiac effusion, atrial fibrillation/flutter, ventricular tachycardia, and acute kidney failure.

In terms of cardiac catheterization procedures, as presented in Table 4, the SS group had the highest proportion of procedures completed within the Diagnosis-to-Wire time of less than 90 minutes (94.95%), and also achieved the highest proportion within <120 minutes (55.10%) drug-eluting stents (DES) appeared to be the predominant choice (78.92%), followed by plain old balloon angioplasty (POBA) and bare-metal stents (BMS) at 17.08% and 8.91%, respectively. The SS cohort led in DES utilization (85.43%), with the UC and CS groups behind at 80.95% and 71.69%, respectively.

One-year follow-up observations, detailed in Table 5, indicated the CS group had the highest follow-up (75.00%) and readmission rate within one year (44.34%), primarily for staged PCI. Mortality rate at one- and two-years post-discharge revealed the highest death rates in the UC group, with the SS group recording the lowest figures. Kaplan–Meier survival analysis was used to evaluate one- and two-year mortality rates across the public health insurance schemes (Figure 2) which revealed significant differences, with both the SS and CS groups demonstrating notably lower mortality rates at one-year compared to the UC group (p=0.0083 and p=0.0446, respectively). Also, the SS group maintained a significantly lower mortality rate at two-years compared to the UC group (p=0.0055).

Utilizing a multiple Cox regression model (Table 6), several predictors are associated with an increased risk of mortality within one-year post-discharge, including age equal to or more than 65 years, Killip scores 3-4, pre-discharge angiotensin-converting enzyme inhibitor administration, occlusion at the left anterior descending artery, and in-hospital pericardial effusion. Moreover, patients covered by the CS insurance demonstrated a decreased risk of 1-year post-discharge mortality.

User

This study represents the first national investigation into the correlation between public health insurance schemes of Thailand and the long-term mortality rates among STEMI patients receiving primary PCI treatment. Our findings reveal that different health insurance schemes have a statistically significant impact on mortality rates. Specifically, the SS group demonstrated the lowest one-year mortality rate at 4.21%, followed by the CS group at 6.47%. Furthermore, a longitudinal analysis extending to two years supported that the SS cohort consistently showed the lowest mortality rate. Additionally, international studies underscore that the entitlement to treatment profoundly influences both the management and outcomes of patients(9-12).

Upon examining the basic characteristic, SS group was found that they had the youngest average age. We observed that patients aged 65 years or older had nearly twice the risk of mortality within one year, which aligns with previous research indicating that age is a significant predictor of mortality(13, 14). When comparing the basic characteristics of patients in other studies, the SS group was found to be younger than the sample groups in other studies(3, 9-11). In terms of comorbidities, the SS patient group had the lowest number of diabetes mellitus which is predictive factors for short-term and long-term mortality(15, 16). Regarding smoking history, a higher proportion of SS patients were smokers. This finding is consistent with observations in smokers with acute myocardial infarction where the paradoxical protective effect of smoking may be attributed to smoking being a confounding variable(17). When assessing the severity at hospital admission using the Killip score other studies have indicated that higher scores are associated with increased risk of mortality in both the short-term and long-term(18), which is consistent with our findings. The SS patient group had a lower proportion of high Killip scores.

Upon further examination of laboratory blood values, SS patient group exhibited the lowest peak creatinine levels. Previous research has established that elevated creatinine levels are indicative of an increased risk of mortality(13). Our study similarly found that patients with acute kidney injury faced a heightened risk of in-hospital mortality. Additionally, the highest levels of LDL in SS group were observed in comparison to other groups. This phenomenon can be elucidated by the LDL paradox, where it has been documented that patients with elevated LDL-C levels during myocardial ischemia experienced reduced mortality and morbidity, likely attributed to younger age and fewer comorbidities(19).

Shortening the treatment delay time significantly influences mortality rates(20). In our research, the SS patient group exhibited the shortest diagnosis-to-wire time, yet, regrettably, this time frame did not appear as a protective factor. Nonetheless, we encourage that reducing pre-hospital time presents a critical opportunity to enhance treatment outcomes and should be prioritized for additional attention and resource allocation(21).

In terms of procedures, SS patients received DES more frequently than BMS, which DES being the preferred choice of stent used in the procedure(22). This results in superior outcomes in terms of reducing the rate of re-occlusion of the stents(6). The final TIMI score does not differ among the three groups, along with the mortality rate in the catheterization room being similar.

Antiplatelet therapy, beta-antagonists, renin-angiotensin system inhibitors, and statins are all associated with enhanced outcomes in both short-term and long-term cardiovascular diseases (5, 6). The proportion of patients receiving antiplatelet therapy and high-intensity Statin has increased compared to previous studies(3). However, nearly half of the patients still did not receive beta-antagonists or angiotensin converting enzyme inhibitors, a continuing issue from previous research(3). Improving the administration of these medications could present an opportunity to reduce morbidity and mortality in STEMI patients.

The comparison between the CS and UC groups, both groups exhibit similar baseline characteristics and treatment received during hospitalization. However, the CS group demonstrates a lower one-year mortality rate. Additionally, being covered under the CS insurance scheme serves as a protective factor against one-year mortality. This can be attributed to the extensive coverage offered by the CS insurance, which grants patients access to treatment at any public hospital, in contrast to the UC and SS schemes that limit treatment to registered hospitals only(8). Moreover, follow-up data indicates that the CS patient group has the highest rate of one-year treatment adherence.

Our research indicates that healthcare schemes play a significant role in influencing mortality rates among patients. Health equity implies the equitable and appropriate access to high-quality health services for all individuals. Also, distributing equity across different regions is crucial(23). The government aims to evolve the health insurance system to augment quality and extend coverage nationwide, particularly through the UC scheme, which covers the majority of the population(24). The assessment of PCI treatment in UC patients has been increasing, resulting in a noticeable reduction in mortality rates(25).

ChatGPT

Here are the paragraphs with American spelling and grammar corrections:

This study represents the first national investigation into the correlation between public health insurance schemes in Thailand and the long-term mortality rates among STEMI patients receiving primary PCI treatment. Our findings reveal that different health insurance schemes have a statistically significant impact on mortality rates. Specifically, the SS group demonstrated the lowest one-year mortality rate at 4.21%, followed by the CS group at 6.47%. Furthermore, a longitudinal analysis extending to two years supported that the SS cohort consistently showed the lowest mortality rate. Additionally, international studies underscore that the entitlement to treatment profoundly influences both the management and outcomes of patients.

Upon examining the basic characteristics, the SS group was found to have the youngest average age. We observed that patients aged 65 years or older had nearly twice the risk of mortality within one year, which aligns with previous research indicating that age is a significant predictor of mortality. When comparing the basic characteristics of patients in other studies, the SS group was found to be younger than the sample groups in other studies. In terms of comorbidities, the SS patient group had the lowest number of diabetes mellitus, which is a predictive factor for short-term and long-term mortality. Regarding smoking history, a higher proportion of SS patients were smokers. This finding is consistent with observations in smokers with acute myocardial infarction where the paradoxical protective effect of smoking may be attributed to smoking being a confounding variable. When assessing the severity at hospital admission using the Killip score, other studies have indicated that higher scores are associated with an increased risk of mortality in both the short-term and long-term, which is consistent with our findings. The SS patient group had a lower proportion of high Killip scores.

Upon further examination of laboratory blood values, the SS patient group exhibited the lowest peak creatinine levels. Previous research has established that elevated creatinine levels are indicative of an increased risk of mortality. Our study similarly found that patients with acute kidney injury faced a heightened risk of in-hospital mortality. Additionally, the highest levels of LDL in the SS group were observed in comparison to other groups. This phenomenon can be elucidated by the LDL paradox, where it has been documented that patients with elevated LDL-C levels during myocardial ischemia experienced reduced mortality and morbidity, likely attributed to younger age and fewer comorbidities.

Shortening the treatment delay time significantly influences mortality rates. In our research, the SS patient group exhibited the shortest diagnosis-to-wire time, yet, regrettably, this time frame did not appear as a protective factor. Nonetheless, we encourage that reducing pre-hospital time presents a critical opportunity to enhance treatment outcomes and should be prioritized for additional attention and resource allocation.

In terms of procedures, SS patients received DES more frequently than BMS, with DES being the preferred choice of stent used in the procedure. This results in superior outcomes in terms of reducing the rate of re-occlusion of the stents. The final TIMI score does not differ among the three groups, along with the mortality rate in the catheterization room being similar.

Antiplatelet therapy, beta-antagonists, renin-angiotensin system inhibitors, and statins are all associated with enhanced outcomes in both short-term and long-term cardiovascular diseases. The proportion of patients receiving antiplatelet therapy and high-intensity Statin has increased compared to previous studies. However, nearly half of the patients still did not receive beta-antagonists or angiotensin-converting enzyme inhibitors, a continuing issue from previous research. Improving the administration of these medications could present an opportunity to reduce morbidity and mortality in STEMI patients.

The comparison between the CS and UC groups, both groups exhibit similar baseline characteristics and treatment received during hospitalization. However, the CS group demonstrates a lower one-year mortality rate. Additionally, being covered under the CS insurance scheme serves as a protective factor against one-year mortality. This can be attributed to the extensive coverage offered by the CS insurance, which grants patients access to treatment at any public hospital, in contrast to the UC and SS schemes that limit treatment to registered hospitals only. Moreover, follow-up data indicates that the CS patient group has the highest rate of one-year treatment adherence.

Our research indicates that healthcare schemes play a significant role in influencing mortality rates among patients. Health equity implies the equitable and appropriate access to high-quality health services for all individuals. Also, distributing equity across different regions is crucial. The government aims to evolve the health insurance system to augment quality and extend coverage nationwide, particularly through the UC scheme, which covers the majority of the population. The assessment of PCI treatment in UC patients has been increasing, resulting in a noticeable reduction in mortality rates.

User

Cost-effectiveness was not presented in this study as some patients were transferred out of the hospital before recovery, making it difficult to track expenses. In searching for mortality data in the civil registration system, information on the cause of death was not provided. The study was conducted on data from only one tertiary care hospital, resulting in limited patient data distribution which may not accurately represent the treatment outcomes in generally. Therefore, further research is needed to collect data at the national level.

ChatGPT

Here's the paragraph with American spelling and grammar corrections:

Cost-effectiveness was not presented in this study as some patients were transferred out of the hospital before recovery, making it difficult to track expenses. In searching for mortality data in the civil registration system, information on the cause of death was not provided. The study was conducted on data from only one tertiary care hospital, resulting in limited patient data distribution which may not accurately represent the treatment outcomes in general. Therefore, further research is needed to collect data at the national level.

User

We found that the one-year mortality rate of patients with STEMI treated with primary PCI in Thailand tends to be lower. Patients using SS scheme had the lowest one-year and two-year mortality rate, followed by patients with CS scheme, which is consistent with the in-hospital mortality rate. The CS schemes and the administration of angiotensin-converting enzyme inhibitors independently serve as protective factors against one-year mortality, while advanced age, a Killip score of 3-4 at hospital admission, occlusion in the left anterior descending artery, and the presence of pericardial effusion independently elevate the risk of one-year mortality. The improvement of the UC scheme may reduce morbidity and mortality in Thailand.

ChatGPT

Here's the paragraph with American spelling and grammar corrections:

We found that the one-year mortality rate of patients with STEMI treated with primary PCI in Thailand tends to be lower. Patients using the SS scheme had the lowest one-year and two-year mortality rates, followed by patients with the CS scheme, which is consistent with the in-hospital mortality rate. The CS schemes and the administration of angiotensin-converting enzyme inhibitors independently serve as protective factors against one-year mortality, while advanced age, a Killip score of 3-4 at hospital admission, occlusion in the left anterior descending artery, and the presence of pericardial effusion independently elevate the risk of one-year mortality. The improvement of the UC scheme may reduce morbidity and mortality in Thailand.
